# Presence of Human DNA on Household Dogs and Its Bi-Directional Transfer

**DOI:** 10.3390/genes14071486

**Published:** 2023-07-21

**Authors:** Heidi Monkman, Bianca Szkuta, Roland A. H. van Oorschot

**Affiliations:** 1 School of Life and Environmental Sciences, Deakin University, Geelong 3220, Australia; 2 College of Science and Engineering, Flinders University, Bedford Park 5042, Australia; 3 Office of the Chief Forensic Scientist, Victoria Police Forensic Services Department, Macleod 3085, Australia; 4 School of Agriculture, Biomedicine and Environment, La Trobe University, Bundoora 3086, Australia

**Keywords:** forensic, DNA transfer, DNA prevalence, DNA recovery, human DNA, dogs

## Abstract

Awareness of the factors surrounding the transfer of DNA from a person, item, or surface to another person, item, or surface is highly relevant during investigations of alleged criminal activity. Animals in domestic environments could be a victim, offender, or innocent party associated with a crime. There is, however, very limited knowledge of human DNA transfer, persistence, prevalence, and recovery (DNA TPPR) associated with domestic animals. This pilot study aimed to improve our understanding of DNA TPPR associated with domestic dogs by collecting and analysing samples from various external areas of dogs of various breeds, interactions with humans, and living arrangements, and conducting a series of tests to investigate the possibility of dogs being vectors for the indirect transfer of human DNA. Reference DNA profiles from the dog owners and others living in the same residence were acquired to assist interpretation of the findings. The findings show that human DNA is prevalent on dogs, and in the majority of samples, two-person mixtures are present. Dogs were also found to be vectors for the transfer of human DNA, with DNA transferred from the dog to a gloved hand during patting and a sheet while walking.

## 1. Introduction

Consideration of the variables influencing the transfer, persistence, prevalence and recovery (TPPR) of DNA has become increasingly relevant to forensic investigations [1,2,3,4]. While several studies have focused on DNA TPPR aspects relating to the interaction that hands have with various types of items and surfaces and the different manners of handling [5,6,7,8], there is very limited information in relation to human DNA TPPR associated with household animals. This is especially relevant for household pets, such as dogs, due to their interactions with and proximity to their human owners. If DNA was found to be readily transferred to and persist on the fur of dogs upon direct contact by a human, this may provide additional avenues of investigation in situations relating to the ownership, possession, interaction, or treatment of these animals. Furthermore, a dog could then potentially be considered a vector for the indirect transfer of human DNA, which could be a relevant factor when assessing the likelihood of DNA findings within a particular case given alternative activities and modes of transfer. For example, just as humans readily transfer DNA within their own environments [9], dogs may also act as vectors for the transfer of DNA from one room to another within a home, or from the hands of one person to the clothing of another, which may be relevant in investigations of various types of crimes.

A recent study by Monkman et al. [10] indicated that the fur of household cats harbors human DNA and that this is mainly derived from their owners. It is thus likely that the fur of other household pets, including dogs, also harbors human DNA. To the authors’ knowledge, there has only been one study thus far that has investigated the recovery of human DNA from dogs. As part of their study, Brower et al. [11] investigated the extent to which human DNA could be recovered from separate samples taken from the muzzle hair and teeth of a dog 2 min after a person handled the muzzle and rubbed the surfaces of the incisor and canine teeth, and again from another dog 15 min after the muzzle/teeth area was handled. Partial human DNA profiles were generated from hair and teeth samples obtained at 2 and 15 min post-handling; however, it was not clear who the source of the DNA was (i.e., the handler, the owner, or other). In another scenario, Brower et al. [11] investigated the potential of obtaining human DNA from the same areas previously targeted (muzzle hair and teeth) both 2 min after saliva was deposited onto the gloved fingers of an individual and then rubbed on the muzzle and incisor and canine teeth of two dogs. Profiling results were either consistent with one or more inconclusive loci or a match (no inconclusive loci) to the saliva donor’s profile. An additional sample taken from the teeth of one of the dogs 15 min after the first sample also provided a profile that matched the saliva donor. Brower et al. [11] also reported the results of two casework scenarios involving dogs, reporting varied results (limited to no further interpretation could be performed).

While the results from the small-scale study by Brower et al. [11] indicate that human DNA can be obtained from some areas of dogs in certain situations, the extent of human DNA TPPR relating to dogs may be dependent on various factors, including the shedder status of the humans that a dog comes into contact with [12,13,14] and the frequency and manner of interactions they have with a dog [15,16]. As such, it is clear that more extensive investigations are required in respect to human DNA TPPR relating to dogs. This pilot study investigates (a) the quantity and origins of human DNA recoverable from different areas of household dogs, (b) how readily human DNA transfers from a hand to a dog during patting, (c) how readily any human DNA present on dogs transfers to a gloved hand during patting, and (d) how readily human DNA transfers from dogs to the floor when walking. Details regarding the dog, household occupants, and the daily activity of, and interactions with, each dog were considered in the interpretation of findings. The results from this study add to our limited understanding of human DNA collection from household pets and their ability to contribute to, and impact, criminal investigations.

## 2. Materials and Methods

### 2.1. Experimental Design

**Figure 1:**
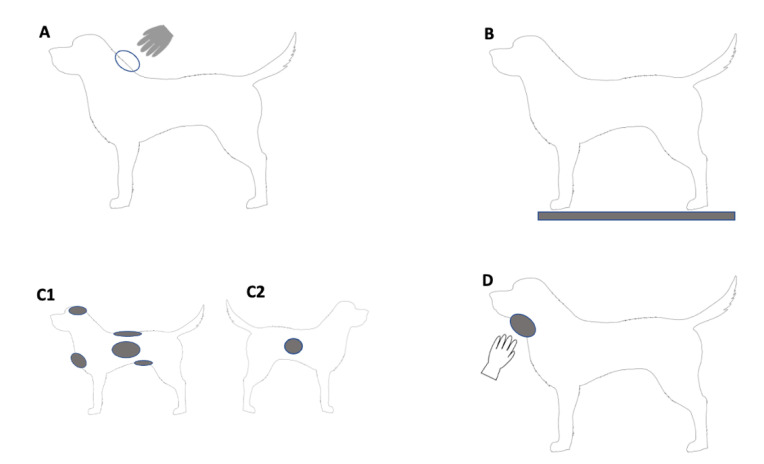
Diagram of the nine areas sampled (shaded grey), including (**A**) a gloved hand after patting the dog’s neck, (**B**) a sheet walked over by the dog, (**C1**) the sites where the dog was swabbed on the chest, top of the head, back, left side and stomach (**C2**) the site where the dog was swabbed on the right side, and (**D**) the area patted with an ungloved hand.

A total of 20 dogs, each from a separate household, were selected based on their friendly nature and ability to adapt to new people and contacts, as advised by the owners (termed the participants). The researcher attended the house of each participant where a total of nine DNA samples were collected directly from the participant’s dog and specific items it came into contact with (Figure 1).

The nine samples were collected in the following order:

1. Upon first entering the house, the researcher patted and scratched the dog on the top of its neck/scruff area with a gloved hand and a sample was collected from the glove to determine if any human DNA that was present could be transferred from the dog to another surface upon contact (Figure 1A). The gloves (InControl, Tarragindi, Australia) were taken directly from a box prior to patting and were assumed to be DNA-free. To ensure consistency across all dogs, they were scratched 4 times (hand opening and closing was considered one scratch) and then patted in the same area a further 4 times.
2. A fresh pair of gloves were donned by the researcher and a sterile plastic sheet (90 cm × 90 cm, Multigate, Villawood, Australia) with a grid-like pattern was adhered to the floor using tape in an area identified by the owner as being suitable. The owner then enticed the dog to walk over the plastic sheet four times (left to right and then from right to left twice across the sheet) and a sample was collected from areas of the sheet contacted by the paws, as identified by the researcher using the grid. As above, this was to determine if any human DNA that was present could be transferred from the dog to another surface upon contact.
3. After donning new gloves, samples were collected directly from six distinct areas of the dog to assess the general presence of human DNA. The six areas, in order of collection, included the chest, top of the head, back, left and right sides, and stomach (Figure 1(C1,C2)); the swabs applied to the left side were placed under the fur to make contact with the skin; all other samples were taken from the surface of the fur. The differing samples from the left and right sides were collected to assess if there was a difference in the quantity of DNA retrieved from the base of the coat/skin surface compared to the top of the fur. Collecting the skin sample from the other side of the dog, rather than the same side as the coat sample, was done to avoid any potential impacts that taking each sample may have had on the other. While care was taken to collect samples from similarly-sized areas from each dog, due to the differences in the size of the dogs and the inability to use a template, there were some differences in the size of the areas swabbed between dogs.
4. After removing gloves, the researcher patted and scratched the dog under the neck/chin area with a single bare hand (Figure 1D) to determine if their DNA could be transferred and detected on a dog during a single contact event. Here, the same scratch/pat method was applied as previously described when using a gloved hand. The area was swabbed immediately after contact.

In addition to the samples from the dog, buccal swabs were obtained from the dog owner(s) and all other residents of the household, with informed consent, to generate reference profiles. The primary carer of each dog was defined as the ‘main human’ (MH) within each household and other household members were designated as Human-2 to Human-5 (or H2 to H5) as applicable, in no specific order; it should be noted that being designated the MH did not necessarily correlate with the amount of time spent with the dog; another person in the household may have had the most contact with the dog. The MH also completed a questionnaire on behalf of the household about the human participants’ living conditions, the dog (breed, hair length, hair shedder type), its habits and interactions with its environment (frequency of walking, contact with household members, the most recent contacts), and any grooming (brushing, washing). Information on these factors is available in Supplementary Table S2. Some of these factors were considered when interpreting the findings.

### 2.2. Sample Collection and Processing

Sampling was conducted by applying a wet-dry swabbing technique using viscose swabs (Forensic Swab L, Sarstedt, Nümbrecht, Germany). Upon collection, wet and dry swabs were returned to their respective tube bases, which contained a ventilation membrane for self-drying, and placed into a labelled envelope for storage. Air-dried swab tips were excised and placed into tubes (wet/dry swabs constituted one sample) and stored at −20 °C until the commencement of DNA analysis. DNA was extracted using the DNA IQ system (Promega, Madison, WI, USA) to a final volume of 60 μL and quantified with Quantifiler^®^ Trio DNA Quantifiler Kit on an ABIPRISM^®^ 7500 (Life Technologies, Carlsbad, CA, USA) and interpreted using HID software. Amplification was carried out using the PowerPlex^®^21 system (Promega, Madison, WI, USA) for 30 cycles using 0.5 ng of template DNA, or, if the sample concentration was below 0.033 ng/μL,15 μL of the sample was used. Amplified product detection and sizing was performed on a 3500 xL Genetic Analyser (Life Technologies, Carlsbad, CA, USA) with an injection voltage of 1.2 kV and an injection time of 24 s. GeneMapper^®^ ID-X software (v1.5, Life Technologies, Carlsbad, CA, USA) was used for genotyping, with an analytical threshold of 175 RFU, as per laboratory protocol.

### 2.3. DNA Profile Interpretation

DNA profiles generated in this study were interpreted using the methods described by Szkuta et al. [8] regarding assigning the minimum number of contributors (MNC) to profiles, use of statistical software STRmix™ (v2.4.5, ESR, Porirua, New Zealand) to deconvolute profiles, and capping the reporting of assigned likelihood ratios (LRs) to 100 billion. In addition to these methods, and for the purposes of this study, profiles containing ≤6 alleles were considered as having limited profiling information (or LIM) and profiles with no allelic peaks present were categorised as providing no profile (or NP); these types of profiles were not assigned a MNC or interpreted further.

Profiles deconvoluted in STRmix™ were also screened against a database containing both participant and investigator references according to the methods described in Reither et al. [9], and STRmix™ mixture proportions were used to assign major (≥70%) and minor (<70%) contributors in mixed profiles; in profiles with a major contributor, one or more minor contributors could be assigned. Profiles with no major contributors were considered to be composed of near-equal (NE) contributions.

In a small proportion of profiles, the number of contributors assigned to the profile, or the minor component of the profile, was less than the number of genetically related individuals that were observed as possible contributors, for example, a two-person mixture of NE contributions, where the mother, father, and child (who was a consenting adult) were all identified as possible contributors. As per the methods of Szkuta et al. [17], these profiles/components were deemed complex family mixtures. For these profiles, the assigned MNC were considered obsolete, and either the profile or the minor component could not be interpreted further.

### 2.4. Data Analysis

Human DNA presence on dogs was investigated by studying six sites on the dogs including the chest, head, back, left side, right side, and stomach. DNA concentrations (ng/μL) were multiplied by the elution volume (60 μL) to determine the DNA amount (ng) for each sample. As this data contained outliers and was not normally distributed, as assessed by boxplots and Shapiro–Wilk test (*p* < 0.05), separate Kruskal–Wallis H tests were conducted to determine if there were differences in DNA quantity between the six sites sampled and between the 20 dogs. Pairwise comparisons were performed using Dunn’s procedure with a Bonferroni correction for multiple comparisons. Statistical analyses were conducted using IBM^®^ SPSS^®^ Statistics v29.

In addition to statistical analyses, general trends regarding the DNA amounts recovered both within and between dogs are reported. Given the preliminary nature of the study and, as such, the limited range of the dataset, it was not possible to perform statistical analyses on the interactions between other variables that may influence DNA yield (e.g., hair length, shedding ability).

General trends in DNA quantities and profiling outcomes were also considered when investigating the transfer of human DNA both to and from the dogs. This included samples that were obtained from dogs after being patted by an ungloved hand, gloves after being used to pat the dog, and sterile sheets after being walked on by the dogs.

## 3. Results

### 3.1. Presence of Human DNA on Dogs

#### 3.1.1. DNA Quantities

**Figure 2:**
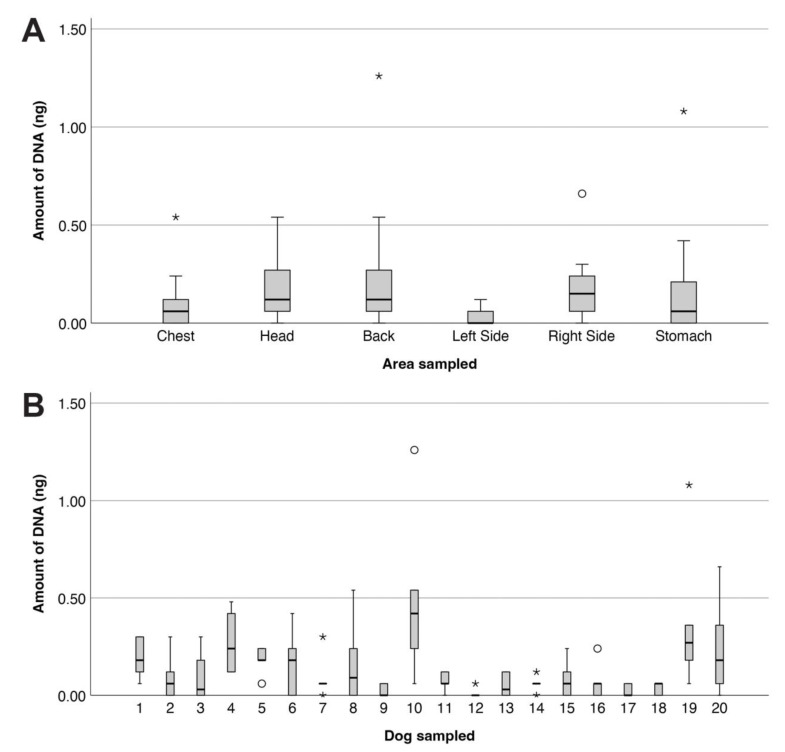
Distribution of DNA quantities (ng) across the six sites sampled from the 20 dogs. Boxplots show the minimum and maximum DNA quantities, along with the median and interquartile range, for (**A**) the six sites sampled and (**B**) each of the 20 dogs. Outliers (*) and extreme outliers (○) are shown.

The general presence of human DNA on dogs was determined from samples obtained directly from six sites on the dogs (Figure 2A). In general, small quantities of DNA were retrieved (Min. 0.000 ng; Max. 1.260 ng; Mdn. = 0.060 ng), with a large number of samples (29.2%) providing no detectable DNA (i.e., 0.000 ng). A Kruskal–Wallis H test was conducted to determine if there were differences in DNA quantity between the six sites sampled. Distributions of DNA quantities were similar for all sites, as assessed by visual inspection of a boxplot (Figure 2A). Median DNA quantities were significantly different between the six sites, χ^2^(5) = 31.331, *p* < 0.001. Post hoc analysis revealed that significantly less DNA was recovered from the left side (Mdn. = 0.000 ng) compared with the right side (Mdn. = 0.150 ng, *p* < 0.001), the head (Mdn. = 0.120, *p* < 0.001), and the back (Mdn. = 0.120, *p* < 0.001).

An additional Kruskal–Wallis H test was conducted to determine if there were differences in DNA quantity between the 20 dogs. As the distributions of DNA quantities were not similar for all dogs, as assessed by visual inspection of a boxplot (Figure 2B), mean ranks are presented. The mean ranks of DNA quantities were significantly different between the 20 dogs, χ^2^(19) = 53.112, *p* < 0.001. Post hoc analysis revealed significantly less DNA was recovered from dog 12 (23.83) compared with dog 10 (101.25, *p* = 0.014), dog 4 (95.58 *p* = 0.046) and dog 19 (95.33, *p* = 0.048), and from dog 9 (29.67, *p* = 0.048) and dog 17 (29.67, *p* = 0.048) compared with dog 10.

#### 3.1.2. Profile Interpretation

Of the 120 samples collected to assess human DNA presence, the MNC could not be determined in 32.5% of profiles generated; 16 provided no profile and 23 yielded limited profiling information (Table 1). These 39 profiles were correlated with samples of low DNA quantities (0.000–0.060 ng), with the majority generated from the left side (14 samples), followed by the chest and stomach (9 samples each) (Supplementary Table S1). A further 5.8% of profiles were considered complex mixtures in their entirety (5 profiles) or solely the minor component (2 profiles) (Table 1). The MNC was assigned in the remaining 61.7% of the 120 profiles obtained, consisting of predominantly two-person mixtures (49 profiles), followed by profiles of a single source (18), and three-person mixtures (7) (Supplementary Table S1).

In 15.0% of the 120 profiles obtained, a single contributor was observed (Table 1). In these profiles, the main human was the most common contributor (9 profiles), followed by an unknown source (6) or another person within the household (3). In a further 19.2% of profiles, a major contributor was observed. This component frequently corresponded to the main human (11 profiles), followed by another person from the household (9) or an unknown source (3). In the 11 profiles where the major contributor was the main human, the minor was generally an unknown source (8 profiles). Where the major contributor was another person in the household, the minor contributor was also another person within the household (4 profiles), an unknown source (4), or both (1). Where the major contributor was an unknown source, the minor was also always unknown (3 profiles). The remaining 29.2% of profiles were mixtures of near equal contributions (Table 1). In general, contributors to these profiles included the main human along with another person from the household (10) or an unknown source (12 profiles), one or more individuals from the household, with or without contributions from an unknown source (8), or only an unknown source (5).

Contributions from unknown sources were observed in 39.2% of the 120 profiles generated: 6 profiles as a single-source contributor, 3 as a major and a minor, 25 as a near equal and 13 as a minor (Table 1). Where there was an unknown single source or major and minor contribution, samples were obtained from the stomach (3 profiles), the head and chest (2 profiles each), and the back and left side (1 profile each). Where near equal, samples were obtained from the head and back (6 profiles each), right side (5) stomach (4), chest (3), and left side (1). Where minor, samples were obtained from the right side (5 profiles), back (3), head and stomach (2 profiles each), and chest (1) (Supplementary Table S1).

#### 3.1.3. Influence of Activities

The information collected regarding the dogs’ characteristics, living conditions, and their interactions with household occupants (Supplementary Table S2) provides the opportunity to consider potential impacts of these on the DNA profiles obtained (Supplementary Table S1).

In general, the contributions to DNA profiles were not reflective of the number of house occupants. For example, dog 8 was from a household of four individuals, though the six profiles generated from samples taken to assess DNA presence were generally single-source (2 profiles) or contained limited to no profiling information (2 profiles). A similar trend was observed for dogs 3 and 12, which were from five- and four-occupant households, respectively, though ≥50.0% of the samples did not produce a profile. In contrast, dogs 17 and 18 were from single-occupant households and most of the profiles were single-source (2 profiles per dog) or contained limited to no profiling information (3–4 profiles per dog).

It was hypothesised that DNA contributions to dogs may be impacted by the amount of time the house occupants spent with the dogs. However, looking again at dogs 3, 8, and 12, the average amount of time each of the house occupants spent with these dogs per day (average 1.9 h per person) was not substantially different compared to that of other dogs and house occupants (average 4 h per person). Further, the owner of dog 15, who spent the most time with their dog (13 h a day), was only observed in three profiles obtained directly from the dog, while the main human spent an average of 6.5 or 5 h a day with dogs 1 and 5 respectively, and their DNA was observed in all six profiles generated from each dog.

Another factor that was considered to impact the presence of DNA was how recent, in relation to sampling, the dogs had been contacted by house occupants. The main human, who was known to regularly contact their dog, and was generally the last person to contact the dog before sampling (on 15 of 20 occasions), was observed in 35.8% of the 120 profiles generated. In addition, eight dogs had been contacted by another house occupant within 10 min of sampling and a single-source profile of the person who made contact was obtained from an area of known contact. While there appears to be many consistencies in the recency of contact by an individual and their contribution to the profiles obtained, there were many occasions where the profile composition was less reflective of the persons making contact. For example, on six separate occasions, single-source profiles from an unknown source were obtained from dogs that were known to have been regularly and recently contacted by people within the household.

There were three dogs (dog 9, 12, and 17) that provided profiles containing limited or no profiling information from four or more of the six sample sites (Supplementary Table S1). Beyond the fact that these dogs all slept on their own bed/kennel within the household, there was little consistency in the activities performed by the dogs or the people associated with them. Two of the dogs were German Shepherds (the only two of this breed in the study) that both frequently spent time inside and outside the house, as did the third dog, which was a Kelpie. House occupants interacted with each of the dogs for between 2–8 h a day on average, and although one dog had been washed two days prior to sampling (dog 17), the other dogs had not been washed for at least one month (Supplementary Table S2). As such, it cannot be determined from this data what factors impacted these results.

A further two dogs (dog 2 and 16) had been washed within 48 h prior to sampling (Supplementary Table S2). It was anticipated that dogs that were washed closer to the time of sampling may provide lower amounts of DNA compared with those that were washed less frequently. This did not appear to be the case, with these two dogs providing moderate DNA quantities (relative to other dogs) and interpretable DNA profiles from most sites (Supplementary Table S1). However, given the small dataset, it is not possible to ascertain the impact of washing on DNA recovery.

### 3.2. Human DNA Transfer to Dogs

DNA quantities ranging from 0.000 ng to 0.420 ng (Mdn. 0.090 ng) were obtained from the 20 samples collected from the necks of the dogs after being patted/scratched with a bare hand (Supplementary Table S1). Of the 20 profiles generated, 75.0% could be analysed further, while 50.0% contained DNA of the individual patting the dog (Table 1). This included two profiles as a major contributor (along with an unknown minor), seven as a near equal contribution (along with contributions from other people in the household or unknown sources), and one as a minor contributor (main human major). Where the DNA from the person patting the dog was not observed as a contributor, or the main human (2 profiles) or another member of the household (1) were observed as a major, a near equal mixture of the main human and another person from the household was obtained (1), or only DNA of the main human was detected (1).

### 3.3. Human DNA Transfer from Dogs

#### 3.3.1. To a Gloved Hand upon Contact

DNA quantities ranging from 0.000 ng to 2.040 ng (Mdn. 0.090 ng) were obtained from the 20 samples taken from the gloved hand after patting the dogs (Supplementary Table S1). Of the 20 profiles generated, 65.0% could be analysed further (Table 1). In mixed profiles, the main human (3 profiles) or unknown sources (2) contributed to the major profile, while in profiles of near equal contributions, DNA from various persons were observed, including those from the household but not the main human (1 profile), the main human and an unknown source (1), the main human and another person from the household (1), and only an unknown source (1). Single-source profiles of the main human (1 profile) or unknown sources (3) were also observed (Table 1).

#### 3.3.2. To a Sheet upon Walking

In general, small quantities of DNA (Min 0.000 ng; Max 0.3600 ng; Mdn. 0.000 ng) were obtained from the sheets after being walked over by the dogs (Supplementary Table S1). Many of the samples (11 of 20) yielded no detectable DNA (Supplementary Table S1). Of the 20 profiles generated, 65.0% of profiles could not be analysed further; 4 provided no profile, 9 contained limited information and 1 was a complex family mixture (Table 1). In the remaining mixed profiles, the major contributor corresponded to a person within the household but not the main human (2 profiles) or an unknown source (1), while in the one profile of near equal contributions, a person within the household but not the main human and an unknown source were observed as contributors. Single-source profiles yielding DNA from a person within the household but not the main human (1) or unknown sources (2) were also observed (Table 1).

## 4. Discussion

### 4.1. Presence of Human DNA on Dogs

This part of the study aimed to assess the general presence of human DNA on household dogs. Although the quantity of DNA varied both within the sites sampled on the same dog and between dogs, human DNA was recovered from all 20 dogs in this study. Overall, higher quantities of DNA were recovered from the back, head, and right side of the dog. It was hypothesised that those sites contacted most recently may result in higher quantities of DNA, however, questionnaire data did not show that these three areas were the only ones contacted by the main human most recently before sampling (Supplementary Table S2). For example, the chest had been contacted by the main human up to 10 min prior to sampling on 12 occasions (comparable to the head, which had been contacted on 13 occasions), yet this site provided some of the lowest yields. In fact, all of the areas sampled, as well as the paws, had been contacted by at least one person in the household within 10 min to 2 h prior to sampling.

Samples were taken from both the left and right sides of the dogs, with the sample recovered from the left side taken from deeper in the fur and closer to the skin. Interestingly, this sample consistently provided the lowest quantities of DNA. Given that the left side was contacted by house occupants at a similar frequency to the right side (Supplementary Table S2), it is theorised that DNA collected by dogs is retained on the surface of the fur rather than being embedded under it. The stomach also provided samples with low quantities of DNA that frequently resulted in uninterpretable profiles or profiles from unknown sources (as major or single-source). This area was also taken from areas of skin, as opposed to fur, and was infrequently contacted by house occupants (Supplementary Table S2). Given these slight trends, further research is required to understand the factors impacting the transfer and persistence of human DNA on the top of a dog’s fur versus at the base of the dog’s fur and on its skin.

The amount of time the owners spent with the dogs varied significantly between households and the people within them (Supplementary Table S2). Those that tended to spend more time with the dog also tended to be the last person who contacted the dog before sampling occurred, and those that touched the dog the most tended to be the most prevalent in the DNA profiles generated from that dog. These results are consistent with a study where samples collected from office areas showed that the DNA primarily belonged to the last person who touched the area and/or the person who most frequently used the area [18]. In addition, interactions may change between owner and dog depending on the size of the dog; it has been shown that smaller dogs have more intense contact and, in general, are hugged and kissed more regularly [16]. Further exploration is required to determine what factors in relation to human proximity may impact the amount of DNA and profile types recovered from dogs, and if the interactions between human and dogs of varied sizes have an effect on the amount of DNA and from where it is recovered.

DNA from one or more unknown sources was observed in 40.6% of the 180 samples taken from the dogs and the items they came into contact with, regardless of whether or not the dogs had been contacted by an individual for whom a reference profile was not obtained. However, it is not uncommon to observe foreign DNA in profiles generated from swabbed surfaces. A recent study by Reither et al. [9] collected samples from flooring surfaces within homes and observed DNA from unknown sources i.e., DNA from individuals who did not reside within the home, in 62.0% of samples. In the present study, the potential origin of DNA from unknown sources found on the dogs could include direct deposits from interactions with individuals for whom reference profiles were not obtained, or indirectly from the household occupants or the surfaces within the household.

Another element that was not greatly explored in this study that may have influenced the amount of DNA from various individuals, including unknown sources, recovered from the dogs was the frequency of washing and grooming. It was anticipated that dogs that were washed closer to the time of sampling may provide lower amounts of DNA compared with those that had not been washed recently, as has been demonstrated in humans, where hand washing can have an effect on the amount of DNA recovered [12,19,20]. However, the washing time frames considered in this study did not appear to have a general effect on the amount of DNA recovered, nor on the profiling outcomes (Supplementary Table S2). However, more structured investigations are required to ascertain the impact of washing a dog on the loss of human DNA, and how quickly human DNA accumulates post washing. Other factors that are normally associated with washing that may have an effect, such as type of shampoo and where the dog is washed, may also play a role, along with other factors such as hair length and shedding ability. These aspects could be a focus of future studies. We urge that as further research in this area becomes available, the interactions between the potentially relevant factors be studied in depth. Trends in the MNC and contributors to the DNA profiles obtained were also considered in conjunction with the history of contact with the dog.

### 4.2. Human DNA Transfer to Dogs

This part of the study aimed to assess the extent of DNA transfer from a single known contact with a specific area of the dogs. It was demonstrated that DNA could be transferred from a hand to a dog after being scratched/patted, as described in Section 2.1, with DNA of the individual patting the dog recovered in 10 of the 20 profiles generated. The frequency of DNA transfer observed in this study is consistent with other studies investigating the transfer of DNA from the hand(s) to a surface upon contact [8,21,22]. As the individual patting the dog was not detected as a contributor in half of the profiles obtained, it is reasonable to suggest that the last person to touch the dog will not always be reflected in the profile. This too is consistent with another study in which the depositor was occasionally not observed as a contributor to the DNA profile retrieved from the item contacted [8]. In a further study, the last handler of an item was not always observed as the major contributor [23].

### 4.3. Human DNA Transfer from Dogs

This part of the study aimed to assess the extent of DNA transfer from the dogs to an object upon contact. In general, little consistency was observed between the amount of DNA obtained directly from the dogs and the DNA yield from the gloves and the sheet; the three dogs providing the lowest DNA yields (as determined by mean rank) when sampled directly (i.e., dogs 9, 12, and 17), provided samples with little (0.06 ng) to no detectable quantities of DNA from these contacted items, while others, such as dogs 10 and 4, which provided amongst the highest yields when sampled directly, provided low yields from the contacted items (Supplementary Table S1).

When contacted by a gloved hand, a detectable level of DNA was transferred on 19 of 20 occasions, 13 of which provided a profile that could be analysed further (Table 1). This rate of transfer is similar to other studies looking at DNA transfer from one surface to another upon contact [21,24,25]. In comparison, the areas that were directly swabbed on the dog in similar areas to those in this part of the study (i.e., the head and the back) also had in excess of 13 occasions where profiles that could be analysed further were obtained. Profiles obtained from the gloves also had a very similar mixture of individuals identified from the two closest locations that were sampled directly (i.e., the head and back), which is likely due to their proximity and the way people generally interacted with the dogs.

Compared to the gloved hand, less DNA was obtained from the sheet after the dogs had walked over it. The difference may be due to the paws’ repeated contact with surfaces while walking, resulting in the loss of DNA, as well as the small contact area of the paws, the relative quantities of DNA on the surfaces that paws come into contact with, and/or different rates of DNA persistence on the paws compared to other areas of the dog. In addition, general dirt and grime that dogs might collect when walking around both inside and outside the home, may be collected during sampling and inhibit the extraction of DNA [26,27]. Compared to the area contacted by the gloved hand (i.e., the neck), the origin of the DNA that was recovered from the sheet was likely acquired by the paws from other areas of the house that the dog was walking on, such as flooring and couches, and indirectly transferred to the sheet. Reither et al. [9] found that the DNA of household occupants was found in areas that the person had not occupied, and it was hypothesized that it had been moved there by other occupants. In addition, Thornbury et al. [28,29] showed that DNA can be agitated off substrates, including readily shaken off items such as clothing and towels. Therefore, it is plausible that the human DNA recovered from the sheet may also, in part, have come from DNA containing material falling off the dog whilst walking over the sheet. Further experiments are required to gain additional insights on the transfer of human DNA to and from dogs, and to provide data for the calculation of probabilities given particular casework relevant scenarios.

## 5. Conclusions

This preliminary study has shown that some level of human DNA could be retrieved from all areas of the dogs that were sampled, though some areas consistently provided more DNA than others. In addition, DNA profiles where contributors could be assigned were also obtained from each sample site. While the dog owner(s), and people residing in the same household as them, were generally detected in the DNA profiles, DNA from unknown sources was also observed, and the questionnaire data did not always account for this. This study also demonstrated that human DNA can be transferred to dogs upon contact by a person’s hand and that it can be transferred from dogs to a contacting surface, such as during patting and walking. This information may assist those investigating criminal acts in which dogs are involved to consider situations in which it may be useful to sample for human DNA from a dog. It also showed that investigators may need to consider dogs as a vector for indirect transfer of human DNA within particular scenarios. Further studies relating to the transfer of human DNA to and from dogs are required to build our understanding and provide data that will assist forensic investigators and legal arbiters.

## Figures and Tables

**Table 1:** Summary of DNA profile compositions obtained from the six samples recovered from each of the 20 dogs (DNA presence), the two samples recovered from items coming into contact with each of the dogs (gloved hand and sheet), and the sample recovered from each of the dogs after patting (bare hand). NE—Near Equal, MH—Main Human, H2—Human 2, H3—Human 3, U—Unknown, P—person patting the dog.

Profile Composition	DNA Presence	Transfer from Dog		Transfer to Dog
		Gloved Hand after Contact	Sheet after Walking	Dog after Patting
No profile	16	1	4	1
Limited information	23	5	9	4
Complex family mixture	5	1	0	0
Maj MH + Min Complex	2	0	0	0
Maj MH + Min P + Min U	0	0	0	1
Maj MH + Min 2U	1	1	0	0
Maj MH + Min H2	1	0	0	0
Maj MH + Min U	7	2	0	2
Maj H2 + Min H3	2	0	0	0
Maj H2 + Min MH	1	0	0	0
Maj H2 + Min U	4	0	1	0
Maj H3 + Min H2 + Min U	1	0	0	0
Maj H3 + Min H2	1	0	1	0
Maj H3 + Min U	0	0	0	1
Maj P + Min U	0	0	0	2
Maj U + Min U	3	2	1	0
NE MH + H2	7	1	0	1
NE MH + H2 + U	3	0	0	0
NE MH + U	12	1	0	0
NE H2 + 2U	1	0	0	0
NE H2 + H3	3	1	0	0
NE H2 + U	3	0	0	0
NE H3 + 2U	1	0	0	0
NE H3 + U	0	0	1	0
NE P + H2 + H3	0	0	0	1
NE P + H2 + U	0	0	0	1
NE P + H3	0	0	0	1
NE P + H3 + U	0	0	0	1
NE P + U	0	0	0	3
NE 2U	5	1	0	0
SS H2	0	0	1	0
SS H3	3	0	0	0
SS MH	9	1	0	1
SS U	6	3	2	0

## Data Availability

Data are contained within the article or Supplementary Materials. If required, more information may be made available upon request. No access to DNA profiles will be provided to anyone.

## Supplementary Materials

The following supporting information can be downloaded at: https://www.mdpi.com/article/10.3390/genes14071486/s1, Table S1: raw DNA analysis data; Table S2: raw questionnaire data.
